# Case Report: Morphologically striking eruptive xanthomas with lobulated papules: a sentinel sign of severe metabolic dysregulation

**DOI:** 10.3389/fendo.2026.1764866

**Published:** 2026-02-27

**Authors:** Tiantian Lu, Fuyuan Zhuge, Wei Cai, Xiaofang Zhang, Dajun Lou, Dihua Huang

**Affiliations:** 1 Department of Endocrinology, Shaoxing People’s Hospital, Shaoxing, Zhejiang, China; 2 Department of Dermatology, Shaoxing People’s Hospital, Shaoxing, Zhejiang, China

**Keywords:** cauliflower-like papules, eruptive xanthoma, fenofibrate, insulin therapy, metabolic syndrome, new-onset diabetes, severe hypertriglyceridemia

## Abstract

Eruptive xanthoma (EX) is a rare but clinically important dermatologic manifestation of severe hypertriglyceridemia and often serves as a cutaneous indicator of profound disturbances in glucose metabolism. Here, we describe a 27-year-old man who presented with numerous yellowish papules that clustered into lobulated, cauliflower-like plaques on the trunk and limbs, serving as the first clinical indication of underlying metabolic dysregulation. Laboratory investigations revealed extreme hypertriglyceridemia (60.45 mmol/L) and newly diagnosed diabetes mellitus (HbA1c 14.1%). Skin biopsy demonstrated foamy histiocytes and Touton giant cells in the dermis. The patient received combination therapy with fenofibrate (200 mg/day), intensive insulin therapy, and a low-fat diabetic diet, leading to rapid normalization of triglyceride levels from 60.45 mmol/L to 2.47 mmol/L by Day 32. The cutaneous lesions flattened and progressively regressed in parallel with metabolic improvement, leaving only faint post-inflammatory hyperpigmentation at the final evaluation. This case emphasizes that uncommon lobulated morphology may serve as an early visual clue of eruptive xanthomas, reinforcing the diagnostic value of cutaneous signs as metabolic sentinels and highlighting that early dual-target treatment can prevent life-threatening complications.

## Introduction

Eruptive xanthoma (EX) is a rare but clinically important dermatologic manifestation strongly associated with severe hypertriglyceridemia and insulin deficiency (1). Although often asymptomatic, EX may represent one of the earliest visible signs of profound metabolic dysregulation and can precede the diagnosis of diabetes or other underlying metabolic disorders (2).

The condition results from the intradermal deposition of triglyceride-rich lipoproteins, with histopathology typically showing foamy histiocytes and Touton giant cells (3). Because extremely elevated triglyceride levels significantly increase the risk of acute pancreatitis, timely recognition of EX in dermatology or general clinical settings is crucial (4, 5).

Here, we report a young adult in whom EX served as the first clinical clue to previously unrecognized metabolic derangement. This case highlights the importance of cutaneous findings as sentinel markers of systemic disease and underscores the need for early, mechanism-based evaluation when EX is identified.

## Case presentation

**Figure 1:**
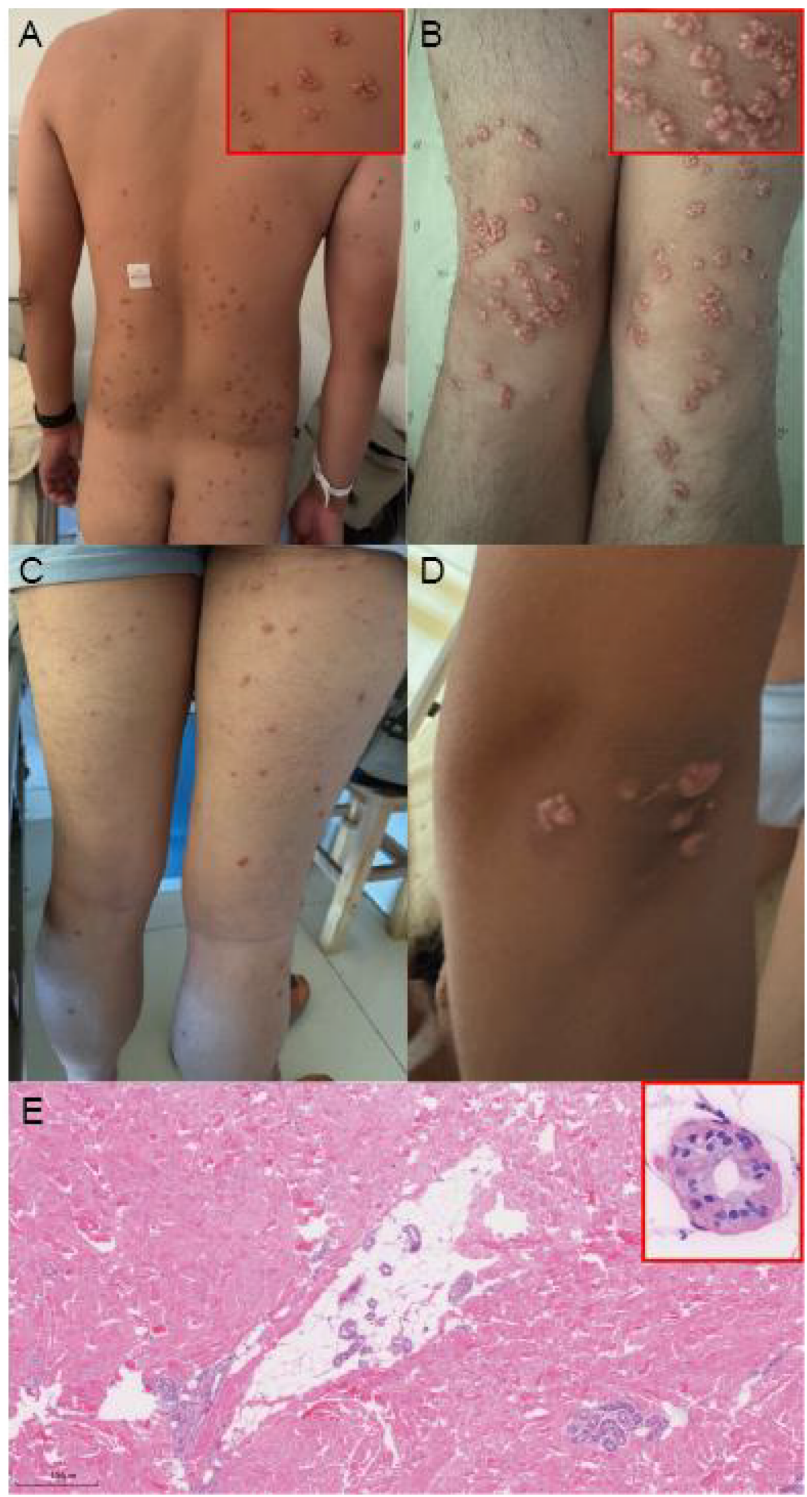
Numerous pink–yellow papules with a lobulated, cauliflower-like surface are distributed across multiple anatomical regions. The lesions are dome-shaped, waxy, and tend to cluster or coalesce into multi-lobular plaques, predominantly over areas with mechanical friction. **(A)** Back and buttocks. **(B)** Bilateral knees. **(C)** Thighs. **(D)** Elbow region. **(E)** Histopathology (H&E): epidermis essentially normal; mid-to-deep dermis showing diffuse, nodular aggregates of foamy histiocytes and Touton giant cells, with scattered lymphocytes and neutrophils and mild stromal fibrosis. An inset at higher magnification highlights characteristic Touton giant cells with ring-arranged nuclei and foamy cytoplasm. Scale bar = 500 µm. The uniform clinical morphology together with characteristic histopathology confirms the diagnosis of eruptive xanthomas associated with severe hypertriglyceridemia.

A 27-year-old man presented to our dermatology clinic with diffuse yellow papules that had begun six months earlier. Lesions involved the back, buttocks, upper limbs, and thighs, occurring in clusters and raised above the skin surface. Notably, across all involved sites, including the knees and other extensor areas, the lesions were exophytic and multilobulated with a verruciform surface, coalescing into raised plaques characterized by a central dell and a prominent peripheral ridge, creating a three-dimensional, cauliflower-like configuration. This morphology differs from previously reported coalescent or plaque-type eruptive xanthomas, in which discrete papules typically merge into relatively flat or mildly elevated plaques rather than forming a distinctly lobulated and outward-growing architecture (**Figures 1A-D**).

He reported polyuria, polydipsia, and dry mouth, and had received no prior treatment. He denied a personal history of dyslipidemia. Regarding the etiology of his condition, the patient explicitly denied any family history of genetic disorders, hypertriglyceridemia, diabetes mellitus, hypercholesterolemia, or premature atherosclerotic cardiovascular disease. He reported no alcohol misuse and no use of medications known to induce hypertriglyceridemia, including systemic corticosteroids, retinoids, estrogen-containing agents, thiazides, beta-blockers, protease inhibitors, or atypical antipsychotics. He had no known allergies and no chronic medication use.

Skin biopsy from a representative papule (**Figure 1E**) showed an essentially normal epidermis and diffuse, nodular cellular aggregates in the mid-to-deep dermis. On high power, there were numerous foamy histiocytes with small round nuclei without atypia and typical Touton giant cells (ring-arranged nuclei with granular/vacuolated cytoplasm). A minor admixture of lymphocytes and neutrophils was present, with mild stromal fibrosis. These findings supported a xanthomatous lesion consistent with EX.

Laboratory evaluation revealed marked hypertriglyceridemia (triglycerides, TG, 60.45 mmol/L), elevated total cholesterol (TC, 15.84 mmol/L) and LDL-C, and reduced HDL-C. Venous blood samples were grossly lipemic (chylous). Liver and renal function were normal. To rigorously exclude secondary causes of hypertriglyceridemia other than diabetes, a comprehensive workup was performed. Thyroid function tests were within normal limits, ruling out hypothyroidism. Evaluation of the pituitary-adrenal axis showed normal cortisol and adrenocorticotropic hormone rhythms, excluding Cushing’s syndrome. Furthermore, screenings for autoimmune liver diseases, antinuclear antibodies (ANA), and tumor markers were all negative.

Glycemic indices were abnormal with HbA1c 14.1% and fasting plasma glucose 14.95 mmol/L, supporting diabetes mellitus. Vital signs were stable. Further evaluation for diabetes classification showed negative results for five islet autoantibodies. A standard oral glucose tolerance test with concurrent insulin and C-peptide measurements showed delayed and attenuated insulin and C-peptide response curves, indicating preserved but not exhausted β-cell function. Anthropometrics showed BMI 27.47 kg/m² and waist circumference 100.5 cm, indicating central obesity. According to the International Diabetes Federation (IDF) criteria, central obesity is required and, in Asian men, is defined as a waist circumference ≥90 cm. In addition to central obesity, the patient met multiple accompanying components, including severe hypertriglyceridemia (TG 60.45 mmol/L), low HDL-C (0.61 mmol/L), and type 2 diabetes mellitus requiring treatment, thereby fulfilling the IDF definition of metabolic syndrome (6). Key laboratory values are summarized in **Tables 1** and **2**.

**Table 1:** Key metabolic biomarkers and supportive laboratory values at baseline and last follow-up.

Parameter	Baseline (Day 0)	Last follow-up (Day 92)	Reference range
Lipid metabolism			
TG (mmol/L)	60.45	0.81	0.56-1.70
TC (mmol/L)	15.84	4.48	2.84-5.69
HDL-C (mmol/L)	0.61	1.25	1.03-1.55
LDL-C (mmol/L)	9.29	2.79	1.55-3.36
Glucose metabolism			
Fasting glucose (mmol/L)	14.95	5.78	3.9-6.1
HbA1c (%)	14.1	NA	4.0-6.0
2-h postprandial glucose (mmol/L)	20.3	NA	3.9-7.8
Clinically relevant laboratory markers			
ALT (U/L)	40.0	45.5	9-50
AST (U/L)	30.0	31.0	15-40
Creatinine (μmol/L)	50.4	65.2	59-104
Lipase (U/L)	59.1	NA	0-67
Amylase (U/L)	45.3	39.2	40-132

**Table 2:** Oral glucose tolerance test with insulin and C-peptide measurements.

Time (min)	Plasma glucose (mmol/L)	Insulin (pmol/L)	C-peptide (pmol/L)
0	15.05	53.53	930.77
30	17.68	119.45	1389.2
60	21.61	127.23	1673.4
120	20.3	91.18	1666.5
180	17.79	76.54	1583.7

OGTT, oral glucose tolerance test. Post-load insulin and C-peptide responses indicate preserved β-cell function.

The patient was diagnosed with hypercholesterolemia with severe hypertriglyceridemia and new-onset type 2 diabetes and was referred from dermatology to endocrinology for further management. Treatment was initiated with fenofibrate 200 mg/day, metformin 1 g/day, acarbose 0.3 g/day, and insulin therapy (initial continuous subcutaneous insulin infusion, followed by premixed insulin aspart 30 after discharge). A low-fat diabetic diet was initiated with dietitian counseling.

**Figure 2:**
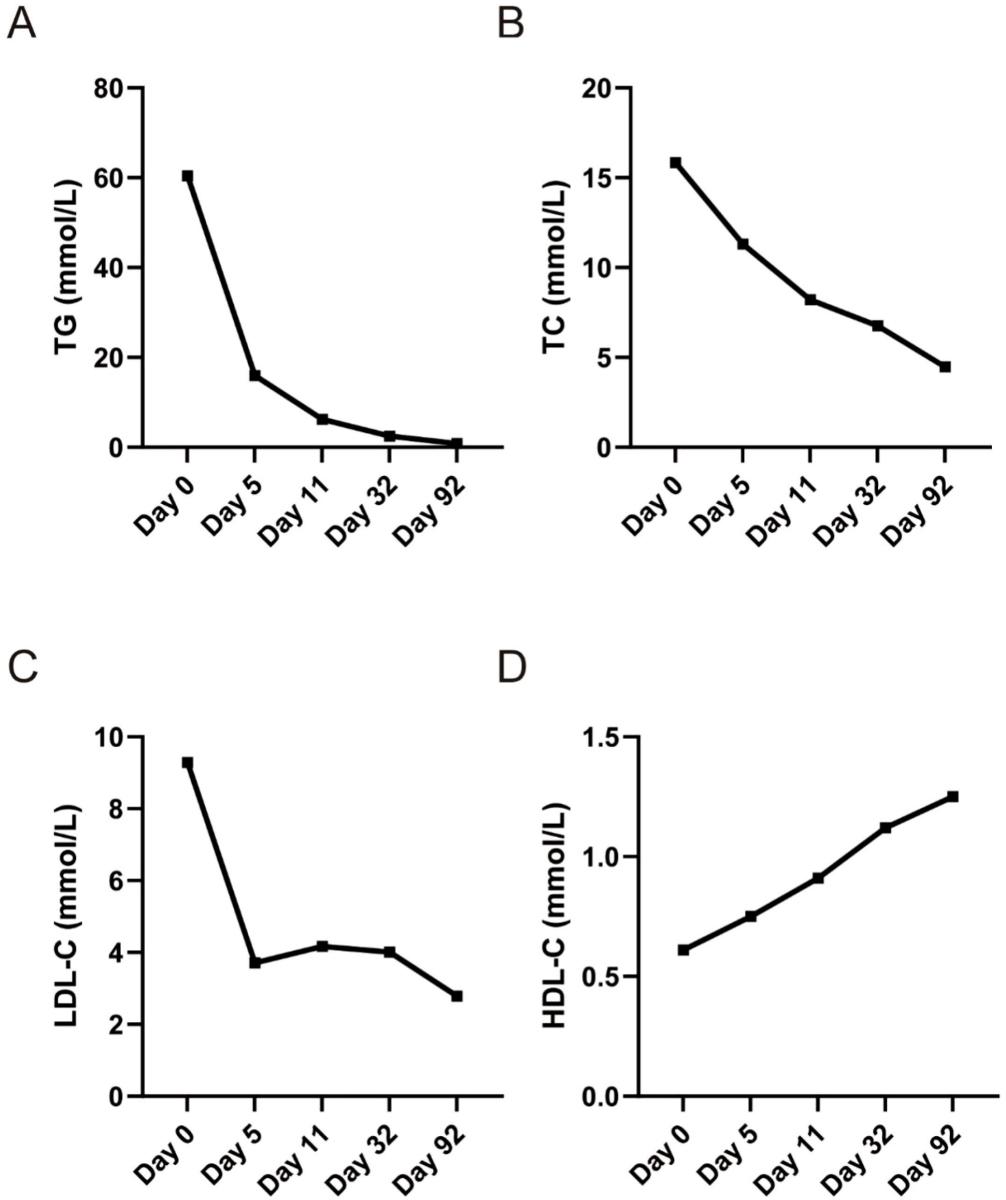
Trends of lipid profile parameters during treatment follow-up. Sequential laboratory measurements were obtained on Day 0, Day 5, Day 11, Day 32, and Day 92 after the initiation of therapy. **(A)** Serum triglycerides (TG) demonstrated a rapid and marked decline during the first treatment week and reached near-normal levels by Day 32. **(B)** Total cholesterol (TC) progressively decreased throughout the follow-up period. **(C)** Low-density lipoprotein cholesterol (LDL-C) showed an initial sharp reduction from baseline, followed by a period of stabilization and a further decrease by Day 92. **(D)** High-density lipoprotein cholesterol (HDL-C) steadily increased over time, reflecting metabolic improvement and lipid profile normalization. HDL-C, high-density lipoprotein cholesterol; LDL-C, low-density lipoprotein cholesterol; TC, total cholesterol; TG, triglycerides.

Serum TG declined rapidly during the first week of treatment and continued to fall progressively throughout follow-up. Levels decreased from 60.45 mmol/L at baseline to 2.47 mmol/L by Day 32 and approached normal values at 0.81 mmol/L by Day 92 (**Figure 2**; **Table 1**). TC and LDL-C also demonstrated steady improvement over time, while HDL-C gradually increased in parallel with metabolic stabilization.

After metabolic stabilization, the therapeutic regimen was adjusted on Day 67 to co-formulated insulin degludec/aspart (IDegAsp) 10 U once daily to simplify the glycemic management regimen, together with metformin 0.5 g twice daily and acarbose 0.1 g three times daily, while fenofibrate was continued.

Given the severe hypertriglyceridemia, the patient was closely evaluated for pancreatitis. He reported no abdominal pain. Serum amylase was within the normal range at presentation, and lipase levels were normal on Day 5; both enzymes remained normal at the final follow-up. Abdominal ultrasonography was performed to screen for visceral pathologies; results indicated hepatic steatosis but revealed a morphologically normal pancreas with no signs of inflammation, edema, or pseudocyst formation. Thus, both acute and subclinical pancreatitis were effectively ruled out.

**Figure 3:**
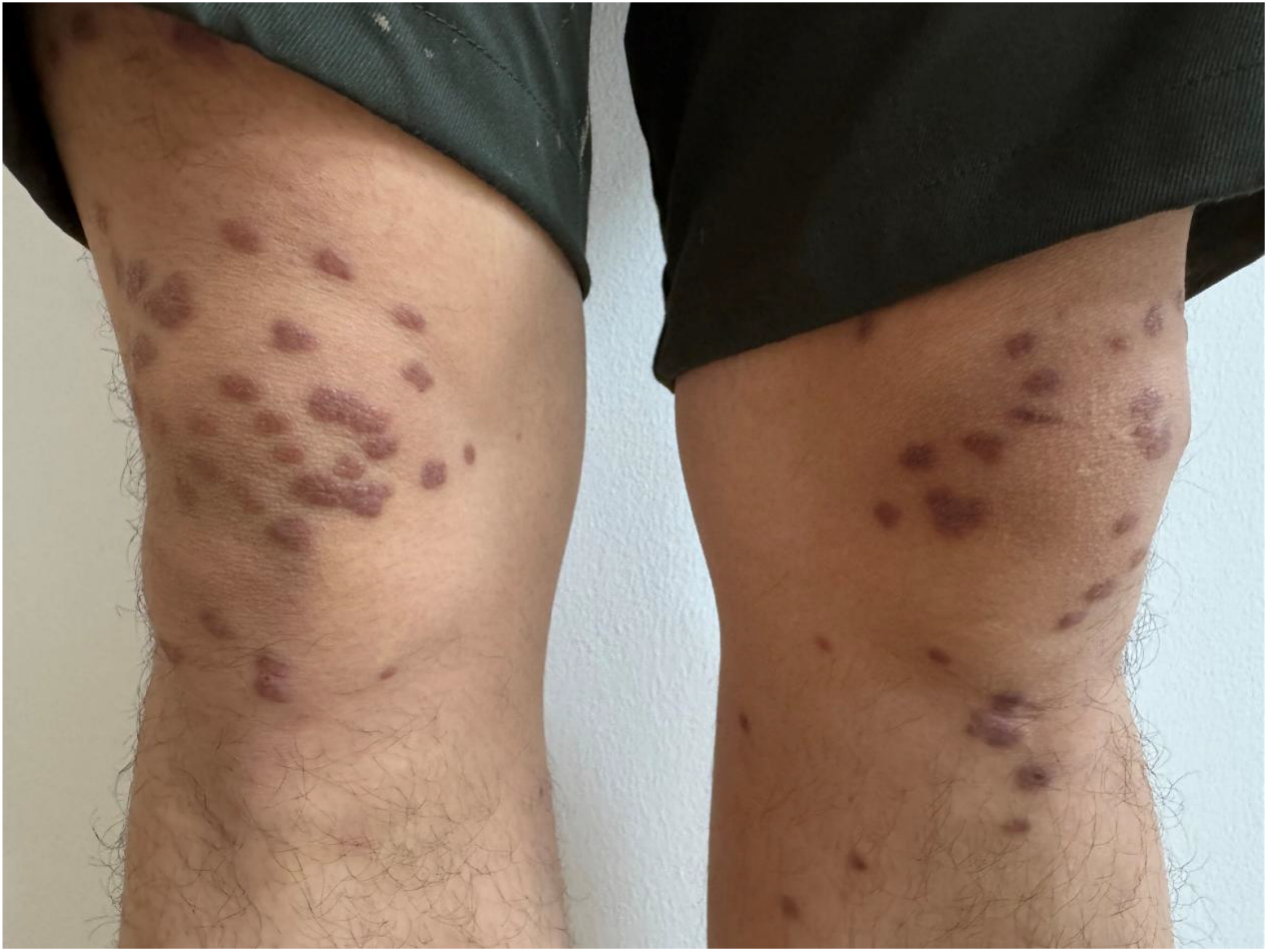
Follow-up appearance of eruptive xanthomas. The papular lesions had fully regressed with only mild post-inflammatory hyperpigmentation remaining and no recurrence.

Cutaneous outcomes mirrored the biochemical response. The papules flattened progressively and fully resolved by the last follow-up visit, leaving only faint post-inflammatory hyperpigmentation without palpable lesions or recurrence (**Figure 3**). Crucially, no recurrence of the xanthomas was observed during the 3-month follow-up period. Metabolic parameters demonstrated sustained improvement; at the final visit, the patient maintained a fasting plasma glucose of 5.78 mmol/L and a triglyceride level of 0.81 mmol/L (**Tables 1**, **2**), indicating stable glycemic and lipid control. The patient reported satisfaction with symptom improvement, and no adverse drug reactions were documented.

## Discussion

In this 27-year-old patient, EX was the initial clue to severe hypertriglyceridemia in the setting of new-onset diabetes. Early treatment targeting lipid and glucose abnormalities was followed by complete cutaneous remission, and no episode of acute pancreatitis occurred.

The eruption was notable for pan-site coalescence into lobulated, cauliflower-like plaques rather than only smooth discrete papules, a pattern occasionally illustrated in image reports but less often emphasized. Clinicopathologic correlation, integrating the extreme TG elevation with characteristic foam-cell/Touton histology, was particularly helpful given the atypical verruciform–lobulated morphology and the broader clinical differential diagnosis (7).

Clinically, EX typically presents as sudden-onset crops of yellow to erythematous papules on extensor surfaces and buttocks, often in the context of marked hypertriglyceridemia (8, 9). When lesions adopt a verruciform, lobulated, or confluent appearance, as observed in our case, the clinical differential diagnosis extends beyond classic eruptive xanthomas and includes entities such as verruciform xanthoma, which is typically localized and papillomatous (10); molluscum contagiosum, characterized by umbilicated papules (11); sebaceous hyperplasia, presenting as soft yellow papules with a central dell, most often on the face (12); and condyloma acuminatum, which usually manifests as verrucous papules or plaques in the anogenital region (13). Additional mimickers of yellowish papules may include xanthogranuloma and other xanthomatous disorders. In this clinical setting, histopathological examination plays an important role in establishing the diagnosis, particularly given the atypical verruciform and lobulated morphology and the need to exclude infectious or verrucous mimickers. In our patient, biopsy provided clinicopathologic confirmation by demonstrating foamy histiocytes and Touton giant cells in the dermis.

Histopathologic studies in EX have demonstrated dermal deposition of triglyceride-rich lipoproteins, including chylomicron remnants, which are phagocytized by dermal macrophages, leading to foam cell formation and sometimes Touton giant cells (14). Mechanistically, insulin deficiency and/or insulin resistance dampen lipoprotein lipase (LPL) activity while increasing hepatic very-low-density lipoprotein (VLDL) production, favoring the circulation of triglyceride-rich lipoproteins. Additional inhibition of LPL by molecules such as ANGPTL3, ANGPTL4 and ANGPTL8 further impairs lipolytic clearance, promoting chylomicronemia (15). Thus, the accumulation of chylomicrons and VLDL remnants in the dermis provides the substrate for xanthoma formation. The rapid clinical resolution after metabolic correction underscores the lipid-driven, rather than structural, nature of these lesions.

Importantly, the presence of residual pigmentation should not be misinterpreted as persistent disease activity. Post-inflammatory hyperpigmentation is a recognized sequela in EX and reflects dermal remodeling after lipid clearance rather than ongoing inflammation or metabolic instability (16, 17).

Treatment that restores LPL-mediated clearance (insulin) and reduces VLDL production (fibrates), together with fat restriction, is recommended in very high TG to limit pancreatitis risk (5, 18, 19). High cutaneous response rates with early combination therapy, whereas the role of topical anti-inflammatory or skin-directed therapy remains uncertain and appears limited compared with systemic metabolic correction (20). In our patient, clinical flattening closely paralleled the biochemical improvement, particularly during the first month of treatment, a practical bedside proxy that therapy is on target.

The probability of acute pancreatitis rises markedly above ~1000 mg/dL (11.3 mmol/L) (21, 22). When symptoms persist or TG remain very high, brief hospitalization for insulin infusion and supportive care is reasonable. Therapeutic plasma exchange (TPE) has been traditionally established as an adjunctive treatment for hypertriglyceridemia-associated acute pancreatitis (23–25). However, emerging case-based evidence suggests that TPE may also be considered as a rescue option in selected patients with severe xanthomatosis refractory to optimized conventional therapy, with reports of rapid triglyceride reduction and improvement of debilitating cutaneous lesions (26). In the present case, in contrast to refractory courses requiring invasive intervention, the patient demonstrated a rapid biochemical response to insulin-based therapy combined with fibrates and dietary fat restriction. Given the prompt decline in triglyceride levels and the absence of pancreatitis, plasma exchange was not pursued, supporting intensive medical management as the first-line approach in responsive cases.

This atypical cauliflower-like morphology expands the known phenotypic spectrum of eruptive xanthomas and underscores that clustered, verruciform or lobulated yellow papules should prompt immediate metabolic evaluation even before laboratory confirmation. Three points are practice-relevant: young-onset EX as the presenting sign, a coalescent lobulated pattern across multiple sites that broadens the clinical spectrum, and paired imaging with serial triglyceride data that clarifies the clinicochemical trajectory under mechanism-based therapy. Limitations include the single-patient design and lack of lesional immunophenotyping or genetic testing, which were not clinically indicated given the rapid response.

## Conclusion

This case demonstrates that EX can serve as an early visible marker of profound metabolic dysfunction, particularly severe hypertriglyceridemia associated with previously unrecognized diabetes. Prompt recognition and mechanism-based therapy led to rapid metabolic stabilization and complete regression of cutaneous lesions, with only transient post-inflammatory hyperpigmentation remaining. Awareness of EX as a dermatologic sentinel finding may facilitate earlier diagnosis, reduce the risk of life-threatening complications such as acute pancreatitis, and improve clinical outcomes.

## Data Availability

The original contributions presented in the study are included in the article/supplementary material. Further inquiries can be directed to the corresponding authors.
